# Quality of Life After Orthognathic Surgery in Swedish Patients: A Register‐Based Cohort

**DOI:** 10.1002/cre2.942

**Published:** 2024-08-28

**Authors:** Eric Johansson, Bodil Lund, Martin Bengtsson, Mikael Magnusson, Lars Rasmusson, Magnus Ahl, Bo Sunzel, Mats Sjöström

**Affiliations:** ^1^ Department of Specialist Dentistry, Oral and Maxillofacial Surgery Colloseum and Smile AB Taby Sweden; ^2^ Department of Dental Medicine Karolinska Institute Stockholm Sweden; ^3^ Medical Unit of Plastic Surgery and Oral and Maxillofacial Surgery Karolinska University Hospital Stockholm Sweden; ^4^ Department of Oral and Maxillofacial Surgery The University Hospital of Skåne Lund Sweden; ^5^ Department of Oral and Maxillofacial Surgery, The Sahlgrenska Academy University of Gothenburg Gothenburg Sweden; ^6^ Maxillofacial Unit Linköping University Hospital Linköping Sweden; ^7^ Department of Oral and Maxillofacial Surgery The Institute for Postgraduate Dental Education Jönköping Sweden; ^8^ Department of Oral and Maxillofacial Surgery Public Dental Health Växjö Malmö University Malmö Sweden; ^9^ Oral and Maxillofacial Surgery Umeå University Hospital Umeå Sweden; ^10^ Department of Odontology Umeå University Umeå Sweden

**Keywords:** ortognathic surgery, quality of life, questionnaire

## Abstract

**Objective:**

This study aimed to evaluate the effect of orthognathic surgery on quality of life among Swedish patients.

**Materials and Methods:**

Patients subjected to orthognathic surgery due to dentofacial deformity (DFD) and registered in the National Register of Orthognathic Surgery (NROK) in Sweden between 2017 and 2020 were eligible for inclusion in this study. The Swedish‐validated Orthognathic Quality of Life Questionnaire (S‐OQLQ) was used to evaluate patient quality of life before and after surgery. The S‐OQLQ measured each patient's subjective experience regarding social aspects.

**Results:**

Eighty‐four participants were included in this cohort study, including 45 men (mean age 24.7 years), 48 women (mean age 23.4 years), and eight patients who stated no gender. Women generally graded several aspects of the S‐OQLQ higher than men, including facial aesthetics *p* = 0.029), oral function (*p* < 0.001), and awareness of facial deformity (*p* = 0.0054). For all domains of the questionnaire (social, facial aesthetics, function, and awareness), a significant improvement was seen 6–24 months after surgery (*p* < 0.001). Women rated improvement of function and awareness of facial deformity higher than men (*p* < 0.001 and *p* = 0.039, respectively).

**Conclusion:**

Quality of life aspects of orthognathic surgery have a strong impact on the treatment outcome. Although functional impairment is often considered a major indication for surgery, the social and aesthetic influence of DFD is highly rated by patients, whereas pain is not an issue before or after treatment.

## 1 Introduction

Dentofacial deformities (DFDs) can affect patients' masticatory function and speech but may also influence their social life and self‐esteem, leading to decreased self‐confidence for the individual patient (Silva et al. 2016). Patients with DFDs are less satisfied with their appearance compared to patients with normal dentofacial features, and the nature and severity of the condition affect patients differently (Johnston et al. 2010). Besides functional problems, DFD patients are more prone to having headaches and temporomandibular symptoms (Abrahamsson 2013). The patients' main concerns for seeking treatment are aesthetics, function, and low self‐confidence (Cunningham, Hunt, and Feinmann 1995; Modig, Andersson, and Wårdh 2006; Øland et al. 2011).

A combination of orthodontic treatment and orthognathic surgery is normally required to correct DFD. The surgery includes a variety of different procedures to correct discrepancies in the facial skeleton and dental arches (Reyneke 2022). Orthognathic surgery techniques have been used worldwide for over five decades and are currently routine in oral and maxillofacial surgery and generally associated with low morbidity and complication rates (Jędrzejewski et al. 2015; Panula, Finne, and Oikarinen 2001).

A systematic review evaluating the effect of orthognathic surgery on patient's health‐related quality of life (HRQoL) from an international perspective showed that, although few studies remain after quality assessment, meta‐analyses support a positive impact on quality of life (QoL) (Meger et al. 2021). Knowledge gaps in orthognathic surgery have been identified (Österberg et al. 2017). To support the value of orthognathic surgery, large‐scale data on patient satisfaction and treatment quality are required (Österberg et al. 2017). In 2017, a national web‐based quality register was initiated for Swedish orthognathic surgery (NROK) (Sjöström et al. 2022). The overall aim of NROK has been to improve the quality of care by evaluating indications, pre‐ and postoperative procedures, length of hospitalization, complications, treatment outcome, equality of care, and patient satisfaction. In Sweden, healthcare institutions are expected to provide open data regarding the rates of success and adverse events and incentives for embracing the concept of patient quality registers. Furthermore, decision‐makers demand that given healthcare be evaluated using quality registers and that key data are open and publicly available. The requirements on the outcome of treatment and healthcare have increased in recent years and are expected to be audited for quality and evidence. Measures of the psychosocial perspective and patient perception are important in evaluating whether the treatment is successful and appropriate (Song and Yap 2017). Solid evidence of patient benefit from treatment is a basic requirement for financing care with public means. Furthermore, research should aim at higher levels of evidence in study design, making it possible to quantify the changes for different types of DFDs and treatments (Soh and Narayanan 2013; Liddle et al. 2015; Duarte et al. 2022b). By understanding the patient's perception of treatment, the quality of the planned treatment can be improved (Choi et al. 2010; Kufta et al. 2016). Therefore, a validated HRQoL questionnaire translated into the language spoken by the patient cohort of interest will further increase the quality of the evaluation.

The aim of this study was to evaluate the effect of orthognathic surgery on HRQoL for patients registered in NROK.

## 2 Methods

### 2.1 Study Design

This study is a prospective pseudo‐anonymized survey study with a register‐based cohort design approved by the Swedish Ethical review authority in Sweden (Dnr 2020‐05609).

### 2.2 Ethics Approval and Consent to Participate

Ethical approval was obtained before the onset of the study (Dnr 2020‐05609). This study was conducted in accordance with the Declaration of Helsinki regarding medical protocol and ethics. Consent was collected from participants before inclusion according to Swedish legislation on quality register.

### 2.3 Consent for Publication

All patients were informed about the study and gave their consent by answering the Swedish‐validated Orthognathic Quality of Life Questionnaire (S‐OQLQ).

### 2.4 Study Population

This study is a pilot test for the usefulness of S‐OQLQ and consisted of a cohort of patients (*n* = 101) registered in NROK and subjected to orthognathic surgical procedures at four Swedish oral and maxillofacial units (Umeå, Uppsala, Jönköping, and Linköping) between December 2017 and June 2020. No registrations were made about the specific surgical procedure. Patients with no postoperative Orthognathic Quality of Life questionnaires (OQLQs) were excluded from the study (*n* = 17).

### 2.5 Data Collection

A validated questionnaire, the Swedish version of the OQLQ (S‐OQLQ), was used. It is sub‐scaled into four main domains for evaluation: social aspects of deformity; facial aesthetics; oral function, and awareness of facial deformities (Cunningham, Garratt, and Hunt 2000; Cunningham, Garratt, and Hunt 2002; Ahl et al. 2020).

### 2.6 Assessment of Quality of Life

All patients were asked to fill out the S‐OQLQ preoperatively before orthodontic treatment and at least 6 months postoperatively after completion of postoperative orthodontic treatment. The items/questions provided answers to the patient's subjective experience regarding changes in social aspects, aesthetics, oral function, awareness of dentofacial aesthetics, chewing, speech, and appearance. The items also gave indirect answers to the patient's assessment of pain.

Overall, the S‐OQLQ consisted of 22 items regarding these domains (Table 1).

**Table 1:** Items on the OQLQ questionnaire. The patients received the Swedish‐validated Orthognathic Quality of Life Questionnaire (S‐OQLQ). Each item was rated from 0 to 4, where 0 means “the statement does not apply to you or does not bother you at all” and 4 means “bothers you a lot.” The total score ranges from 0 to 88, with a higher score indicating poorer quality of life.

1. I am uncomfortable with the appearance of my teeth.
2. I have problems biting.
3. I have problems chewing.
4. There are things I avoid eating because of problems with my bite.
5. I don't like eating in public places.
6. I sometimes get pain in my face or jaw.
7. I don't like seeing a side view of my face.
8. I spend a lot of time studying my face in the mirror.
9. I spend a lot of time studying my teeth in the mirror.
10. I don't like being photographed.
11. I don't like being filmed.
12. I often notice other people's teeth.
13. I often notice other people's faces.
14. I am uncomfortable with the way my face looks.
15. I try to cover my mouth when I meet new people.
16. I feel nervous when I meet new people.
17. I worry that people will make hurtful comments about my appearance.
18. I feel insecure in a social context.
19. I don't like smiling when I meet people.
20. My appearance makes me depressed sometimes.
21. I sometimes think that people are staring at me
22. Comments about my appearance upset me, even when I know that it is not meant seriously.

The preoperative questionnaire was sent to the patient's home address after enrollment and was completed at home without supervision. The completed questionnaire was then sent back to the responsible surgeon for registration in NROK. The baseline questionnaires were completed by the patient with a follow‐up questionnaire at the planned postoperative follow‐up at least 6 months postoperatively. The 6‐month follow‐up was decided after analyzing the postoperative follow‐up for all Swedish surgical clinics, where we realized that the timing varied greatly.

### 2.7 Data Analysis

The participants were anonymized by assigning a non‐identifiable number in which the first figure defined the department of patient registry and the following number was a consecutive number in order of enrollment. The patients' answers were divided into two groups: baseline and follow‐up.

### 2.8 Statistical Analysis

Isolated missing values were handled after discussion by a group including a statistician. In this discussion, three options were identified:

1. Individuals with one or more missing values in a domain would be excluded from that domain.
2. Missing values would be set to 0.
3. Missing values would be extrapolated to the mean value for the replies within the domain in question.

Option 3 was chosen. To ensure that this would not skew data, a first rudimentary control on how this would affect the before and after results was performed. This showed no difference between the methods of choice.

A nonresponse analysis was carried out using the nonparametric analyses Fisher's exact test (binary variables) and Fisher's permutation test (other variables) comparing the 84 individuals with response after surgery to the 17 without a response. The same tests were used when comparing men and women. Differences in the OQLQ before and after orthognathic treatment were tested using the nonparametric Fisher's test for pair comparisons. The age intervals were used when testing the impact of age on baseline OQLQ and differences in OQLQ. Each age interval was tested versus every other with Fisher's permutation test. Two‐sided p‐values were used, and *p* < 0.05 was considered significant.

## 3 Results

### 3.1 Included Patients

A total of 101 patients from four different clinics were included (*n* = 45 men, *n* = 48 women, and *n* = 8 no gender given). Seventeen patients did not respond to the follow‐up questionnaire and were excluded, leaving data from 84 patients available for analyses. Data regarding age were missing for 18 individuals. Among the other 66 patients, the mean age was 24.0 years (SD 8.1, range 18–51). The 37 male patients were somewhat older, with a mean age of 24.7 years (SD 8.1, range 18–51) compared to 23.4 (SD 8.1, range 18–47) for the 39 women, but this difference was not significant (*p* > 0.30).

### 3.2 QoL Outcome After Surgery

A total of 57 of 84 patients had positive changes after surgery in all categories on the questionnaire and 11 in all categories except awareness of facial deformity.

For all four domains of the questionnaire (social, aesthetics, function, and awareness), the overall summary of the items within all domains showed significant improvement at follow‐up 6–24 months after surgery (*p* < 0.001, Table 2).

**Table 2:** Number of individuals and the mean grading at baseline and follow‐up.

		*n*	Mean	SD	Range		*p*
Baseline	Social aspects	101	8.56	7.30	0.0	28.0	
	Facial aesthetics	101	10.52	5.12	0.0	20.0	
	Function	101	9.00	5.17	0.0	20.0	
	Awareness of facial deformity	101	5.63	4.09	0.0	15.0	
Follow‐up	Social aspects	84	2.88	5.12	0.0	26.0	
	Facial aesthetics	84	3.88	4.51	0.0	19.0	
	Function	84	2.41	3.41	0.0	17.0	
	Awareness of facial deformity	84	3.61	3.76	0.0	16.0	
Baseline minus follow‐up	Social aspects	84	5.14	7.40	−26.0	26.0	< 0.001
	Facial aesthetics	84	6.33	5.28	−6.0	20.0	< 0.001
	Function	84	6.61	5.11	−6.0	18.8	< 0.001
	Awareness of facial deformity	84	1.96	3.61	−5.0	12.0	< 0.001

Missing values were randomly distributed among the different domains without a significant difference between the 84 patients with baseline and follow‐up evaluations compared to the 17 patients without follow‐up evaluations according to the four domains at the start (*p* > 0.10), age (*p* = 0.091), or sex (*p* > 0.30).

**Figure 1:**
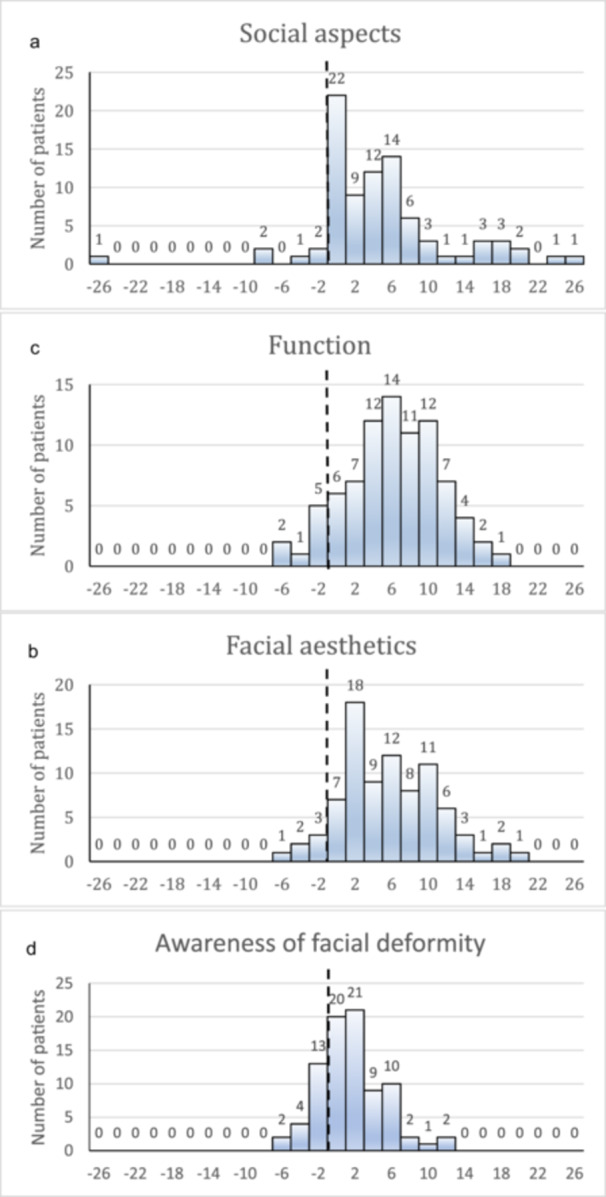
(a–d) Distribution of value differences comparing baseline with follow‐up.

A few individuals reported deterioration at follow‐up with regard to social aspects (*n* = 6), facial aesthetics (*n* = 6), and function (*n* = 8). Nineteen patients reported increased awareness of facial deformity at follow‐up. The differences between baseline and follow‐up had a normal distribution for function, facial aesthetics, and awareness of facial deformity, whereas the results for social aspects were more scattered (Figure 1a–d).

The functional domain consists of five items (Table 1). Four of these questions concern chewing and biting function, which were rated relatively high preoperatively (mean 7.41) and showed strong improvement after surgery (mean 1.51). In contrast, the fifth functional question regarding pain was low before surgery (mean 1.64) and only showed a minor change (mean after surgery 0.89).

### 3.3 Gender Aspects in QoL

**Figure 2:**
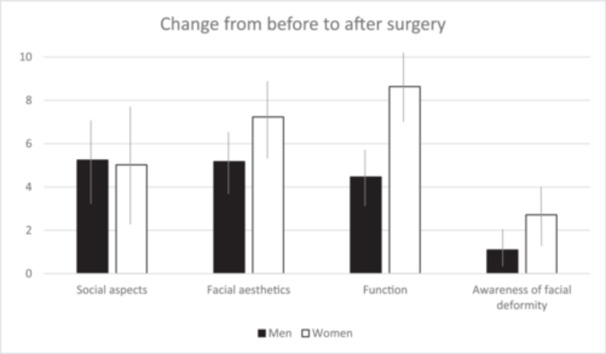
Improvement after surgery in the different domains in relation to gender. Thin lines describe confidence intervals (CI).

One patient of the included population (*n* = 84) did not report on sex, leaving 83 patients for analysis of gender aspects (41 men and 42 women). In ratings before surgery, women graded QoL significantly higher than men with regard to facial aesthetics (*p* = 0.029), function (*p* < 0.001), and awareness of facial deformity (*p* = 0.0054). Regarding social aspects, there was no gender difference before surgery (*p* > 0.30). There was a nonsignificant tendency for women to evaluate the improvement in facial aesthetics higher than men (*p* = 0.072). Women rated improvement of function and awareness of facial deformity considerably higher than men (*p* < 0.001 and *p* = 0.039, respectively; Figure 2).

### 3.4 Influence of Age

#### 3.4.1 Baseline Registrations

Social aspects were judged less beneficial in the two younger age groups, 18–19 (*n* = 25) and 20–29 years (*n* = 30) than in the older age group of 30–51 years (*n* = 11; *p* = 0.25). The opposite tendency was observed regarding function (*p* > 0.30; Table 3).

**Table 3:** Ratings of the different domains before surgery by age group.

		18–19 years (*n* = 25)		20–29 years (*n* = 30)		30–51 years (*n* = 11)	
		Mean	SD	Mean	SD	Mean	SD
Baseline	Social	8.60	6.78	9.53	7.54	6.36	7.34
	Facial	11.00	4.71	9.72	5.47	10.55	6.12
	Function	8.24	5.21	9.13	4.39	10.45	7.10
	Awareness	5.95	3.66	5.87	4.26	4.09	4.61

#### 3.4.2 Follow‐Up

**Figure 3:**
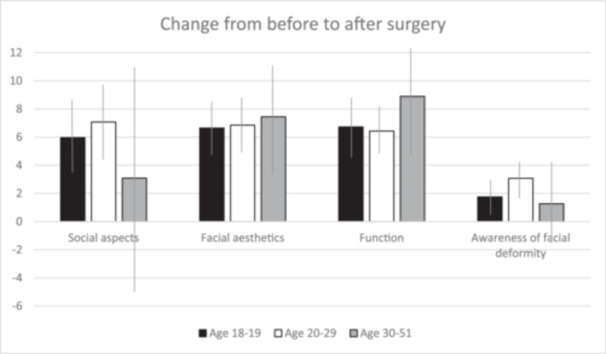
The level of improvement between baseline and follow‐up for the different domains by age group. Thin lines describe confidence intervals (CIs).

There was a tendency that differences in the level of improvement for the different domains to vary between the age groups (Figure 3). For the older age group (30–51 years), the impact on social aspects after surgery was modest and these patients had a tendency to report lower values than the younger age groups (18–19 and 20–29 years). However, this tendency was not significant (*p* = 0.18). The effect on facial aesthetics was evenly distributed between the groups (*p* > 0.30). Although not significant, the impact on function was greater for patients in the age range of 30–51 years (*p* = 0.17).

## 4 Discussion

Impaired function is the key factor in national health service funding of orthognathic surgery in Sweden. It has been acknowledged that orthognathic surgery changes function in a positive way (Al‐Moraissi, Perez, and Ellis 2017; Jung et al. 2018). A decade ago, Ryan, Barnard, and Cunningham (2012) identified the need for a qualitative methodology to complete the full circle of evidence‐based practice. When using questionnaires to evaluate treatment outcomes, it is of utmost importance that the tool has been developed for testing the question at issue and been properly validated. The questionnaire needs to be in the native language of the patient to minimize misinterpretation of questions. To compare and compile international data, translation is required and a specific procedure of back‐and‐forth translation needs to be followed without changing any of the questions, followed by a validation process. Because the S‐OQLQ was developed as a Swedish variant according to a state‐of‐the‐art translation of the OQLQ, data from both these questionnaires can be compared (Song and Yap 2017; Soh and Narayanan 2013). By using this questionnaire, we have a tool not just to complete the full circle of evidence‐based practice in Sweden, but also to provide a stepping stone of compiled international experience with QoL after orthognathic surgery. Larsson, Bondemark, and Häggman‐Henrikson (2021), reported in a systematic review about the increased interest in facial esthetics, especially for patients with orthognathic surgery treatment needs.

To the best of our knowledge, the present study is the first to evaluate QoL using the S‐OQLQ. By using the S‐OQLQ, we have clearly visualized the impact of aesthetics, social aspects, and awareness of facial deformity on QoL in a cohort of Swedish patients referred for orthognathic surgical procedures. The results illustrate the importance of patient benefit beyond functional aspects. The same findings were reported by Tüz et al. (2022). The results from our study highlight the importance that QoL should be considered and weighed in future treatment decisions for patients requiring orthognathic surgical procedures.

The participants in the present study have been followed in the public healthcare system through childhood and adolescence. All children in Sweden have their dental treatment funded by the Swedish dental care system until the age of 23 years. Therefore, an absolute majority of the included patients had their facial development evaluated during growth. The patient cohort in the present study had a mixed socioeconomic background. One can only speculate about the results from a QoL evaluation based on a patient cohort in which aesthetics was the main indication and that every patient would have been seeking treatment on their own initiative. The patient cohort in our study had approximately the same mean ages as reported previously (te Veldhuis et al. 2017; Ver Berne et al. 2022). Our study had the same men/women ratio as the total number of included patients in NROK (Sjöström et al. 2022).

When analyzing the results based on gender, female patients reported that aesthetics and functional aspects were more severe before surgery than the values noted by men. In addition, the subjective outcome in these aspects was greater for female patients after surgery. Scariot et al. (2019) analyzed whether gender and genetic polymorphisms in estrogen receptor alpha (ESR1) and beta (ESR2) are associated with anxiety levels in patients undergoing orthognathic surgery. The authors found that females were more anxious than males at each time point during the study. Our study did not evaluate genetic polymorphisms, but the anxiety level could have affected the female patients' ratings of aesthetics and functional aspects. The importance of facial esthetics was evaluated by Kufta et al. (2016). The authors found that satisfaction with postoperative appearance had the strongest correlation with overall satisfaction. Jung et al. (2018) evaluated the gender‐ and dental education‐specific differences in perception of facial attractiveness for varying ratios of the lower face contour. The authors found that facial images with an increased lower facial height were perceived as being much less attractive. The evaluation was performed by dental students. In our study, the aesthetic aspects were evaluated by the patients themselves; one can speculate that patients who have gone through orthognathic surgery have an increased interest in facial aesthetics. The effect after different surgical procedures may vary, which should be evaluated in future studies.

Patient age was associated with the results in different domains, though not significantly. Therefore, interpretations should be made with caution, and larger patient groups are required to determine whether a difference exists. In light of this, there was a trend that social aspects are less important for older patients (30–51 years) than younger patients. One can speculate that acceptance of DFDs increases with increasing age. Interestingly, there was a tendency for functional aspects to be rated highest in the 30–51 years age group. Impaired function may be more difficult to adapt to or accept later in life, or the impact on functional aspects may increase with age.

Pain does not seem to be a major complaint in this patient group. We could not find any difference in postoperative pain intensity between the sexes. In contrast, Mobini, Mehra, and Chigurupati (2018) found a higher risk of postoperative pain in female patients.

One interesting finding was that 19 patients reported increased awareness of facial deformity after surgery. This can be discussed from two different points. First, the result can be judged to be a positive result of the surgery, as the patient had greater awareness of their facial appearance. On the other hand, the patient assessed their appearance more intensively after a facial change. This has also been discussed by Cunningham et al. (Soh and Narayanan 2013) who suggested that it would be interesting to chart the progress of patients over time to see if respondents eventually show significant improvements in this domain. The change in social aspects did not differ between genders. However, the distribution was more scattered than the other domains. There was also a tendency in the oldest group of patients to have less change in social aspects. Eventually, this lower tendency could be explained by these outliers.

Duarte et al. (2022a) studied patients' oral health‐related QoL (OHRQoL) according to type of DFD and following treatment in a systematic review. The authors found that there is not enough evidence to support differences between Class II and III patients with regard to the OHRQoL impact after orthognathic surgery, but the results indicated less improvement in some domains in Class II patients.

A limitation of the present study was that QoL was not evaluated in light of diagnosis and treatment, and no intra‐reliability test was performed. In addition, the limited number of patients prevented further subgroup analyses. Future studies should consider the influence of general health, including psychological disorders, such as dysmorphophobia and socioeconomic background, on QoL. Campos et al. (2023) reported that planning of aesthetic treatments in the orofacial region should include the patient's perceptions and social context. Alanko et al. (2022) found that oral function and facial aesthetic components were the most stable changes evaluated with OQLQ for patients treated with orthognathic surgery. Future studies should evaluate the long‐term stability of the effect of orthognathic surgery by using the Swedish‐validated Orthognathic Quality of Life Questionnaire (S‐OQLQ).

## 5 Conclusions

This study highlights that the QoL aspects of orthognathic surgery are of utmost importance for the patient‐perceived treatment outcome. Gender seems to have an influence on both expectations and the outcome of treatment, especially regarding function and awareness of facial deformity. Although functional impairment is often considered a major indication for surgery, the social and aesthetic influence of DFDs is highly rated by patients, whereas pain is not an issue before or after treatment.

## Author Contributions

**Eric Johansson:** data collection, project administration, data analyses, writing, reviewing, and editing. **Bodil Lund:** conceptualization, methodology, supervision, analyses of data, writing original draft preparation, reviewing, editing, project administration, and funding. **Mikael Magnusson:** conceptualization, supervision, data analyses, writing, reviewing, and editing. **Mats Sjöström:** writing original draft preparation, supervision, writing, reviewing, editing. **Martin Bengtsson**, **Lars Rasmusson**, **Magnus Ahl**, and **Bo Sunzel:** reviewing and editing. All authors have read the final version.

## Conflicts of Interest

The authors declare no conflicts of interest.

## Data Availability

The data sets generated and analyzed during the current study are not publicly available due to the Swedish Journal Act but are available from the corresponding author on reasonable request.
